# Rapidly Progressive, Symmetrical, Acute Cutaneous Necrosis of Bilateral Lower Limbs: A Rare Presentation of Polyarteritis Nodosa

**DOI:** 10.7759/cureus.58649

**Published:** 2024-04-20

**Authors:** Indika Wettasinghe, Shiran Puthra, Hemal A Sugathapala, Suresh Mendis

**Affiliations:** 1 Internal Medicine, Colombo South Teaching Hospital, Colombo, LKA

**Keywords:** foot drop, cutaneous necrosis, vasculitis, polyarteritis nodosa, medium vessel vasculitis

## Abstract

Acute cutaneous necrosis is a rare presentation of polyarteritis nodosa (PAN). In this study, we report a presentation with symmetrical cutaneous necrosis of the lower limbs, which ascended upward at a rapid rate. A 47-year-old man presented with a fever of one day and pain in the feet for six days. He had no history of claudication. Upon examination, he was febrile, and subtle bluish discoloration was observed on the sole of his foot. There was a bilateral stocking-type paresthesia up to the ankle joint. His blood pressure on admission was 210/120 mmHg. Eight hours later, the pain subsided, but a left-sided foot drop was noted along with the paresthesia extending up both feet to approximately 10 cm above the medial malleolus. The feet turned black, and dark discoloration spread rapidly upward over the next 16 hours, and the skin became necrosed. A clinical diagnosis of vasculitis was established, and the patient received IV methylprednisolone at a daily dosage of 1 g for three days, effectively stopping the advancement of necrosis. This was followed by treatment with IV cyclophosphamide. A conclusive diagnosis of PAN was made, and the patient underwent wound debridement. After three months of physiotherapy, a successful skin graft was performed. Prompt identification of the underlying etiology is crucial to prevent the advancement of necrosis and save the limbs. When vasculitis is suspected, ruling out infectious causes is essential before starting early immunosuppressive treatment.

## Introduction

Acute cutaneous necrosis has multiple etiologies including warfarin-induced skin necrosis, heparin-induced skin necrosis, calciphylaxis, pyoderma gangrenosum, embolic phenomena, and purpura fulminans [1,2]. However, bilateral symmetrical progressive necrosis is very rare and hardly documented. Here, we present a patient who presented with rapidly progressive, symmetrical, acute cutaneous necrosis of the bilateral lower limbs managed as vasculitis and later diagnosed as polyarteritis nodosa (PAN). While cutaneous manifestations such as livedo reticularis, purpura, and ulcers are common presentations of PAN, bilateral acute cutaneous necrosis has been hardly documented [3].

## Case presentation

A 47-year-old male from Moratuwa, Sri Lanka, presented with bilateral lower limb pain in the calf, feet, and toes for six days and fever for one day, along with chills and rigors. He presented because of worsening of pain, which was severe at rest and worsening with movement. He had a history of swelling and pain in the small joints of his hands three weeks back, which had resolved within a week with analgesics. His upper limbs were not affected, and there he had no prior history of lower limb pain or claudication. He reported no urinary or respiratory symptoms, and his bowel movements were normal. However, there he had one episode of vomiting before admission but no abdominal pain.

The patient's past medical, surgical, and allergy history was not significant. The patient was a carpenter and a father of two, and he had no history of IV drug abuse or unsafe sexual behavior.

On examination, he was febrile. His blood pressure was 210/120 mmHg, and his pulse rate was 100 bpm. There were no murmurs. The lungs were clear, the abdomen was soft, and the genitalia was normal. There were no femoral or renal bruits. An examination of the lower limbs revealed no swelling, warmth, or redness, but a bluish discoloration was observed in the sole of the foot. The dorsalis pedis arterial pulse was good. On the motor examination of the lower limbs, hip and knee joint movements had a power of 5/5, but the ankle joint could not be assessed because of pain. Lower limb reflexes were normal. There was a bilateral stocking-type paresthesia up to the ankle joint. There was no tenderness along the spine.

**Figure 1:**
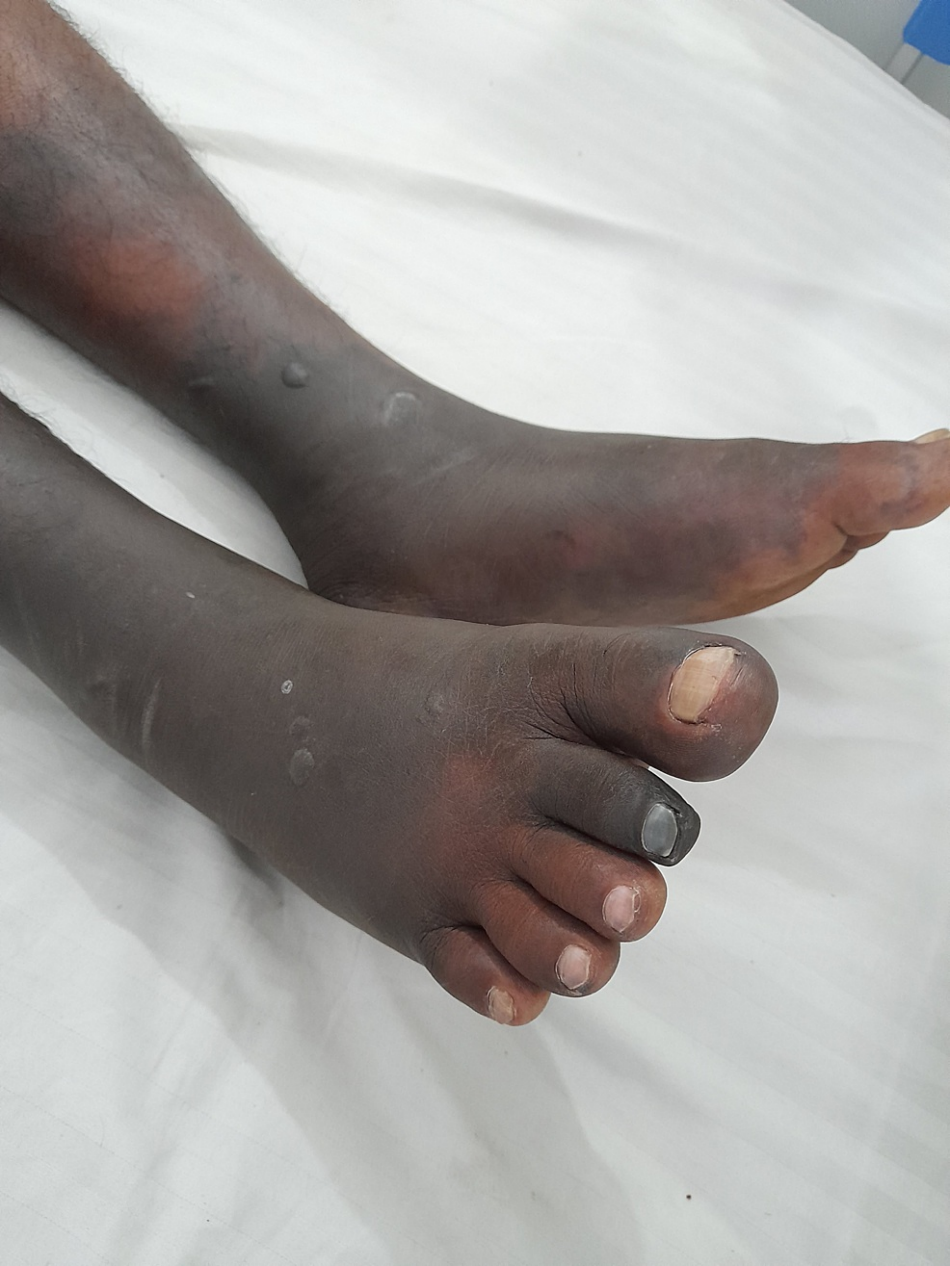
Acute cutaneous necrosis of the bilateral lower limbs

**Figure 2:**
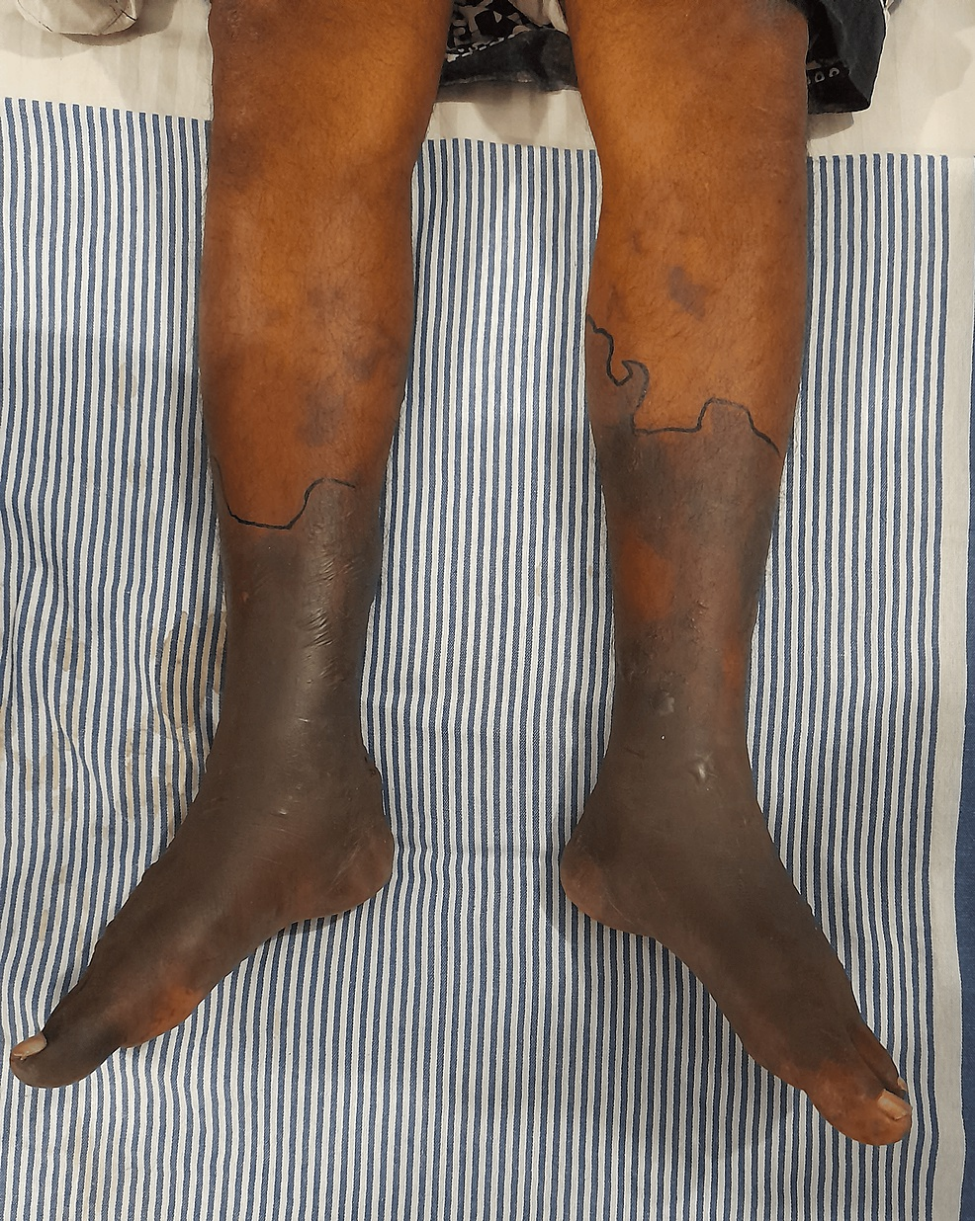
Acute cutaneous necrosis of bilateral lower limbs after the progression of necrosis had halted

Eight hours later, the pain had settled, and a foot drop was noted in the left foot. Paresthesia ascended up in both feet up to 10 cm above the medial malleolus. The feet were black in color, and it looked as if the skin was necrosed (Figure 1). The blackish discoloration ascended up the limbs at a rapid rate over the next 16 hours (Figure 2). CBC, serum creatinine, and electrolytes were normal (Table 1), while the erythrocyte sedimentation rate (ESR) was 45 mm/hr and C-reactive protein (CRP) was 104 mg/L. The chest X-ray was normal, and the abdominal ultrasound and renal artery Doppler showed no abnormalities. On the second day of admission, there was mildly increased proteinuria as evidenced by a urine protein to creatinine ratio (UPCR) of 62 mg/g (< 30 mg/g). However, the repeat UPCR reports were normal, and no dysmorphic red cells were detected in the urine.

**Table 1:** The biochemical parameters of the patient WBC: white blood cells; RBC: red blood cells; HCT: hematocrit; MCV: mean corpuscular volume; CV: corpuscular volume; MCH: mean corpuscular hemoglobin; MCHC: mean corpuscular hemoglobin concentration; RDW: red cell distribution width; SD: size distribution; AST: aspartate aminotransferase; ALT: alanine aminotransferase; INR: international normalization ratio; APTT: activated partial thromboplastin time; DAT: direct antiglobulin test; CRP: C-reactive protein; ESR: erythrocyte sedimentary rate; RBS: random blood sugar; mIU: milli-international units; TSH: thyroid-stimulating hormone

Test	Normal range	26/08/2022	28/08/2022
WBC (×10^9 ^)	4.0-10.0	13.81	23.28
Neutrophils (×10^9 ^)	2.0-7.0	12.56	18.72
Lymphocytes (×10^9 ^)	1.0-3.0	1.0	2.79
Neutrophils %	50-70	90.8	80.3
Lymphocytes%	20-40	7.3	12.0
Monocytes (×10^9 ^)	0.2-1.0	0.17	1.69
Eosinophils (×10^9 ^)	0.02-0.5	0.04	0.06
Basophils (×10^9 ^)	0.02-0.1	0.04	0.02
Monocytes %	3.0-12.0	1.3	7.3
Eosinophils %	0.5-5.0	0.3	0.3
Basophils %	0.0-1.0	0.3	0.1
RBC (×10^12 ^)	4.5-5.5	4.03	3.34
Hemoglobin (g/dl)	12.0-16.0	11.7	9.7
HCT (%)	37-54	35	27.7
MCV (fL)	83-101	86	82.9
MCH (pg)	27-32	28.9	29.9
MCHC (g/dl)	31-34	33.4	35.0
RDW-SD (fl)	11.6-14.0	13.8	42.3
RDW CV (%)	39.0-46.0	43	14.0
Platelets (×10^9 ^)	150-400	208	227
Renal function tests			
Sodium (mmol/L)	136-146	128.7	130.4
Potassium (mmol/L)	3.5-5.1	3.0	4.4
Urea (mmol/L)	2.8-7.2	3.4	4
Creatinine ( µmol/L)	74-110	88.4	82
Calcium(mmol/L)	2.02-2.6	2.35	2.2
Corrected calcium (mmol/L)	2.062-2.60	-	2.59
Phosphate (mg/dcL)	2.9-5.0	-	3.1
Uric acid (µmol/L)	210-420	-	186
Liver function tests			
AST (U/L)	<50	19.3	13
ALT (U/L)	<50	28	90
Total protein (g/L)	66-83	69.12	55.01
Albumin (g/L)	35-52	24.6	26.9
Globulin (g/L )	25-35	44.52	28.11
Total bilirubin (µmol/L)	5.0-21	19.6	9.67
Direct bilirubin (µmol/L)	0.0-3.4	7.16	2.23
Indirect bilirubin (µmol/L)	3.4-12	12.4	7.4
Prothrombin time (Seconds)	10.4-13.6	10.8	-
INR	<1.1	0.95	-
APTT (Seconds)	25-35	24	-
DAT		Negative	-
Inflammatory markers			
CRP (mg/dl)	<5	104.7	108
ESR (mm/hr)	<10	45	34
Procalcitonin (µg/L)	<0.07	-	<0.07
RBS (mg/dl)	<200	142	
Creatinine kinase	<171	-	298
TSH (mIU/L)	0.5-4.7	-	0.493
Blood culture	-	Negative	-
Urine culture	-	Negative	-

The clinical diagnosis of medium or small vessel vasculitis was made, and the patient was given IV methylprednisolone 1 g daily for three consecutive days, which halted the progression of necrosis. He was also given nifedipine 20 mg (slow release) every 12 hours. On day five of admission, with a positive antinuclear antibody (ANA) report and negative hepatitis screen (Table 2), the patient was treated with IV cyclophosphamide 500 mg (7.5 mg/kg) every two weeks for three doses. A final diagnosis of PAN was made, and the patient was continued on oral prednisolone 40 mg mane. As cyclophosphamide could not be continued after the third dose because of limited stocks, mycophenolate mofetil (MMF) 1 g mane and 500 mg nocte were added.

**Figure 3:**
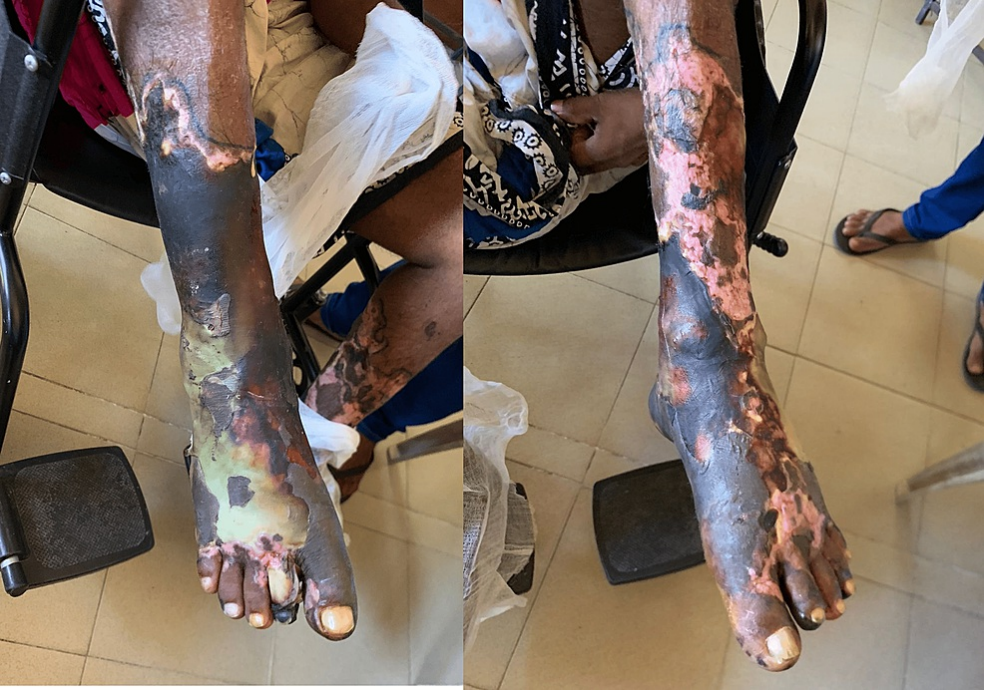
Image of the leg 10 days after admission (before wound toilet) showing blister formation

**Figure 4:**
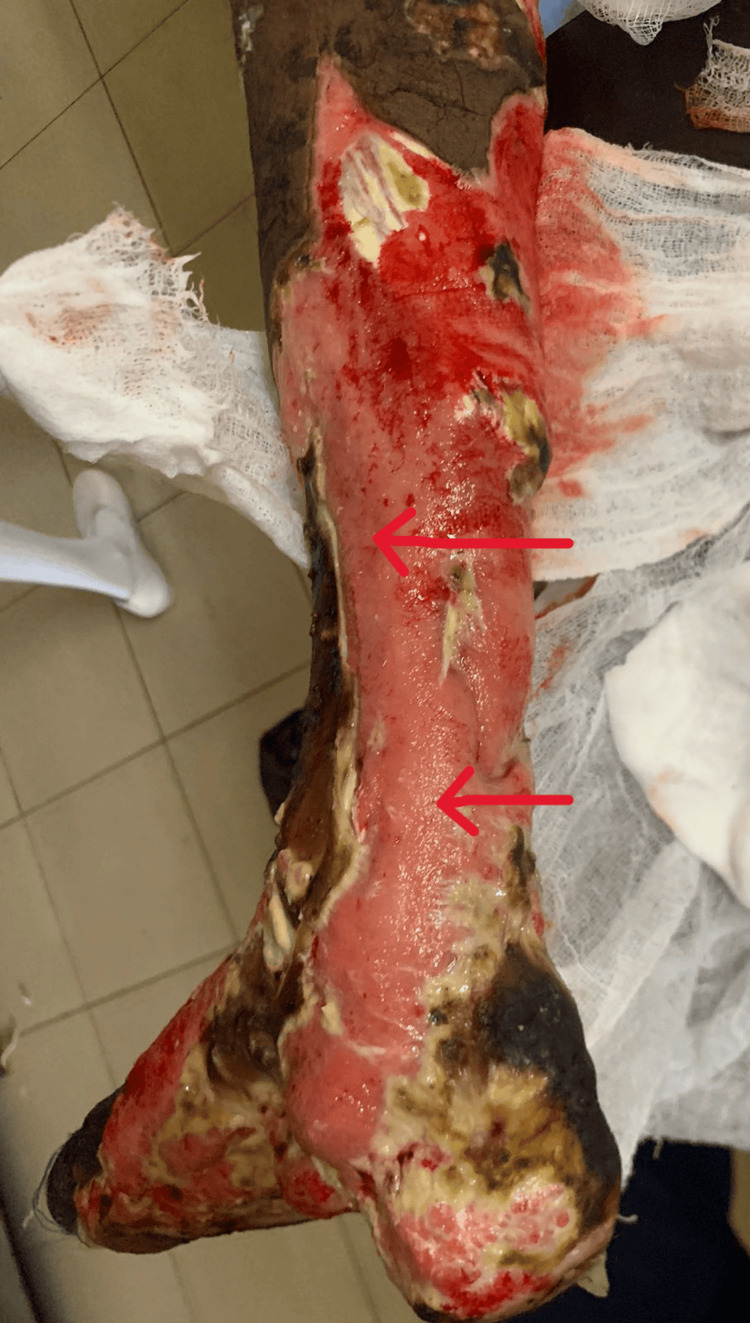
Image of the right lower limb after wound debridement, revealing healthy tissue that was under the necrosed skin

Although the progression of skin necrosis was halted, it was uncertain if the underlying tissue was affected. By day seven of admission, the necrosed skin had formed blisters and started to peel off, and the legs were foul-smelling. The patient was transferred for a vascular surgical opinion, wound debridement was done in stages, and the underlying tissue was found to be viable (Figures 3-4). After three months of physiotherapy and wound dressing, a successful skin graft was done, and the patient regained much of his mobility. From then onward, the patient was followed up monthly, and the doses of MMF and prednisolone were gradually reduced over six months. By the end of six months, the patient was on MMF 250 mg every 12 hours.

**Table 2:** Autoimmune panel and other investigations VDRL: venerology disease research laboratory; HIV: human immunodeficiency virus; ANA: antinuclear antibody; ANCA: antineutrophilic cytoplasmic antibody; C-ANCA: antineutrophil cytoplasmic antibodies; P-ANCA: Perinuclear anti-neutrophil cytoplasmic antibodies

Test	Result
Autoimmune	
ANA-Nuclear pattern	Positive > 1:80
Double-stranded DNA	Negative
C-ANCA	Negative
P-ANCA	Negative
Cryoglobulin	Negative
Rheumatoid factor	Negative
Serum	
VDRL	Negative
HIV	Negative
Hepatitis C antibody	Negative
Hepatitis B surface antigen	Negative

## Discussion

Symmetrical, rapidly progressive, acute cutaneous necrosis of bilateral lower limbs is a rare presentation in clinical practice. It is even rarer for this to be the first presentation of PAN, where the usual clinical manifestations include polyneuropathy (75 %), livedo reticularis/purpura/ulcers (50%), renal disease (50%), gastrointestinal symptoms (40%), and testicular pain (10%) [3].

In recent times, acute cutaneous necrosis has increasingly been documented in patients with COVID-19 infection. Zhang et al. reported seven critical COVID-19 patients presenting with acro-ischemia at a single center in Wuhan [4]. Del Giudice et al. described catastrophic bilateral lower limb necrosis in a COVID-19 patient and noted that the proposed mechanisms were coagulopathy and viral endothelitis [5].

Medium vessel vasculitis, such as PAN, and small vessel vasculitis are two common causes of necrotizing vasculitis. Small vessel vasculitis includes ANCA-associated vasculitis (AAV) (e.g., granulomatosis with polyangiitis, eosinophilic granulomatosis with polyangiitis, and microscopic polyangiitis) and immune complex small vessel vasculitis such as cryoglobulinemia and immunoglobulin A vasculitis [3]. Purpura fulminans and bilateral necrotizing fasciitis can be considered as the other causes of skin necrosis [1].

The differential diagnoses on admission were causes for bilateral lower limb pain, such as vasculitis, peripheral vascular disease, and Raynaud’s phenomenon. With the onset of necrosis, diagnoses such as vasculitis, bilateral necrotizing fasciitis, purpura fulminans, and snake bite were considered.

Even though the dorsalis pedis pulse was good, an arterial Doppler of the lower limbs was done to exclude peripheral vascular causes, and this showed triphasic flow with no evidence of peripheral vascular disease.

The absence of a previous history of fever, the initial slow progression of pain, negative wound swab culture, negative blood and urine cultures, and normal procalcitonin levels were used to rule out the infectious causes. Distinguishing between infectious causes and vasculitis was crucial as the treatment of the latter includes immunosuppressants, which can be detrimental if the former is the cause. Purpura fulminans is an important differential diagnosis wherein there is skin necrosis with disseminated intravascular coagulation (DIC), and it is usually associated with septic shock. The most common infectious causes of purpura fulminans are Streptococcus pneumoniae, Neisseria meningitidis, groups A and B staphylococci, while postinfectious purpura fulminans has been associated with Varicella [2]. In our patient, the coagulation profile and platelet count were normal, excluding DIC, and the blood pressure was consistently high. Given this, and that the blood, urine, and wound swab cultures were negative, purpura fulminans was unlikely.

The diagnosis of PAN was made according to the American College of Rheumatology 1990 Criteria for PAN, where the presence of three or more of the ten criteria has a sensitivity of 82.2% and specificity of 86.6% [6]. Our patient had myalgia, polyneuropathy, and a diastolic blood pressure >90 mmHg on admission. It was not possible to perform a skin biopsy of the affected area and conduct nerve conduction studies because of the necrosis of the skin. The CT aortogram and renal angiogram showed no evidence of vascular aneurysms. Additionally, there was a weight loss of 3.5 kg within two weeks from the time of admission. He had not measured his weight before admission, but he claimed that he had to have lost a lot of weight in the three weeks leading up to the date of admission. The patient regained weight fast, most probably because of prednisolone.

The initial treatment of PAN and AAV is similar. In patients with organ- or life-threatening AAV, recent guidelines recommend inducing remission with either cyclophosphamide and glucocorticoids or rituximab and glucocorticoids [3,5]. According to the CYCLOPS study, pulsed cyclophosphamide caused fewer cases of leukopenia and induced remission at a lower cumulative dose than oral regimens [7]. Although pulsed cyclophosphamide was found to be associated with a higher relapse risk than oral cyclophosphamide, it was not associated with increased long-term morbidity or mortality [8].

For patients with newly diagnosed active, severe PAN, initiating treatment with IV pulse glucocorticoids with cyclophosphamide is recommended over high-dose glucocorticoids alone [9]. One area where the recommendation differs is that in PAN, treatment with cyclophosphamide and glucocorticoids is preferred over rituximab and glucocorticoids [9]. In our patient, a dose of 7.5 mg/kg of IV cyclophosphamide was used, which is lower than the conventional dose. According to a retrospective cohort study consisting of 80 patients, low-dose (7.5-12.5 mg/kg) IV cyclophosphamide resulted in similar relapse rates compared with the conventional regimen (>12.5 mg/kg) and resulted in fewer infections [10]. As the patient did not have life-threatening vasculitis when initiating cyclophosphamide and the fact that there was a significant risk of superimposed infection of the necrosed skin, a lower dose of cyclophosphamide was preferred for this patient.

In a case report, Mazokopakis et al. highlighted the rapid improvement of cutaneous PAN lesions with hyperbaric oxygen therapy, which also led to a long disease-free interval [11]. Hyperbaric oxygen therapy will deliver dissolved oxygen at an excess of the metabolic requirements. This means that oxygen is delivered even if hemoglobin is absent. This changes the local hypoxic conditions caused by vasculitis and promotes the healing process by increasing fibroblast proliferation and neovascularization [12]. Additionally, the inflammatory response and the neutrophilic infiltration in the medium-sized vessels will be reduced because of the reduction of rolling and adhesion of polymorphonuclear cells in the microcirculation [12].

The best therapeutic approach in severe situations such as this is first to exclude infection (including purpura fulminans associated with meningococcemia or staphylococcal toxic shock syndrome) and then identify the etiology [11]. If vasculitis is suspected, the patient should be immediately treated with IV methylprednisolone and cyclophosphamide, along with calcium channel blockers to improve peripheral circulation [9,13].

## Conclusions

Bilateral, symmetrical, rapidly advancing cutaneous necrosis of the lower limbs is a rare presentation in clinical practice. Prompt recognition of the underlying etiology is crucial to halt the progression of necrosis and save the limbs. Early treatment with IV methylprednisolone and other immunosuppressants is crucial to reduce morbidity and mortality in such presentations because of vasculitis. In such cases, ruling out infectious causes is imperative before initiating high doses of steroids. However, individual assessment and management would lead to better outcomes in patients with unusual presentations of vasculitis.
